# Vertebral fracture prevalence and risk factors for fracture in The Gambia, West Africa: the Gambian Bone and Muscle Ageing Study

**DOI:** 10.1093/jbmr/zjae182

**Published:** 2024-11-07

**Authors:** Kate A Ward, Landing Jarjou, Camille Pearse, Mícheál Ó Breasail, Ramatoulie E Janha, Ayse Zengin, Ann Prentice, Nicola J Crabtree

**Affiliations:** MRC Lifecourse Epidemiology Centre, Human Development and Health, Southampton, SO16 6YD, United Kingdom; Nutrition and Planetary Health, MRC Unit The Gambia at London School of Hygiene and Tropical Medicine, PO Box 273, Atlantic Boulevard, Banjul, The Gambia; Nutrition and Planetary Health, MRC Unit The Gambia at London School of Hygiene and Tropical Medicine, PO Box 273, Atlantic Boulevard, Banjul, The Gambia; MRC Lifecourse Epidemiology Centre, Human Development and Health, Southampton, SO16 6YD, United Kingdom; Department of Medicine, School of Clinical Sciences at Monash Health, Monash University, Monash Medical Centre, Clayton, VIC 3168, Australia; Nutrition and Planetary Health, MRC Unit The Gambia at London School of Hygiene and Tropical Medicine, PO Box 273, Atlantic Boulevard, Banjul, The Gambia; Department of Medicine, School of Clinical Sciences at Monash Health, Monash University, Monash Medical Centre, Clayton, VIC 3168, Australia; Nutrition and Planetary Health, MRC Unit The Gambia at London School of Hygiene and Tropical Medicine, PO Box 273, Atlantic Boulevard, Banjul, The Gambia; MRC Nutrition and Bone Health Group, MRC Epidemiology Unit, Cambridge Biomedical Campus, Cambridge, CB2 0QQ, United Kingdom; Department of Endocrinology, Birmingham Women’s and Children’s NHS Foundation Trust, Birmingham, United Kingdom

**Keywords:** analysis/quantitation of bone, DXA, aging, epidemiology, general population studies, osteoporosis

## Abstract

There are limited data describing the epidemiology of vertebral fractures (VF) from resource-limited settings, where the aging population is growing most rapidly. We aimed to determine the prevalence, incidence, and risk factors for VF in The Gambia, West Africa. The Gambian Bone and Muscle Ageing Study is a prospective observational study in men and women aged 40 yr and over. Rural participants had baseline measurements and plasma samples collected and were followed up 6-8 yr later; urban participants had a single measurement. DXA scans were obtained to assess areal BMD (aBMD), body composition, and VF. Prevalence and incidence were calculated. Risk factors for prevalent and incident fractures were tested using logistic regression, in men and women separately, with and without adjustment for age and BMI. At baseline, 581 individuals (298 women) had useable scans, 214 (127 women) at follow-up. Prevalence of VF was 14.8%. Those with VF were older (65.6(11.2) vs 61.7(12.3) yr, *p* = .01) and had lower aBMD *Z*-scores. For example, in women, a 1 SD increase in femoral neck *Z*-score resulted in a lower risk of having a prevalent VF (OR [95% CI]) 0.51 [0.38, 0.73]. In men, lumbar spine *Z*-scores were predictive of prevalent fracture (0.71 [0.53, 0.97]). The incidence of VF over follow-up was 12.1%. Low BMD and grip strength were associated with the odds of having an incident VF. Given the importance of prevalent VF in predicting future VF and other fragility fractures in other populations, our findings are a major cause for concern. VF prevalence in Gambian older adults is similar to elsewhere, despite fractures not being a perceived issue. Risk factors were like those identified elsewhere, including age, aBMD, and bone resorption. Understanding the impact of these fractures is important in a region where the health of the aging population needs to be prioritized.

## Lay Summary

The aging population is growing rapidly, and most in low- and middle-income countries, leading to inevitable increases in the occurrence of low trauma fractures. In Africa, we know little about fractures and how often they occur in older people. Fractures of the spine are the most common fragility fracture in older people and lead to further increased risk of fragility fracture. Women and men aged 40 yr and above, living in The Gambia, West Africa, were invited to take part and followed for 6-8 yr. Participants received spine images to determine the prevalence of spinal fractures and completed surveys, physical function and muscle measurements, and blood samples to identify risk factors for prevalent (pre-existing) and incident (new) fractures. Of the 581 individuals with useable baseline scans, the prevalence of vertebral fractures was 15%. Areal BMD and age were the strongest factors associated with VF. Of the 214 participants with longitudinal scans, the incidence of spinal fracture was 12%. The number of spine fractures in Gambian older adults is similar to elsewhere in the world; this is despite fragility fractures not being considered an issue in African adults. Risk factors were like those identified elsewhere. Understanding the impact of these fractures is important in a region where the health of the aging population needs to be prioritized.

## Introduction

The prevalence and risk factors for vertebral fractures (VF) vary among populations, and there are limited data from resource-poor settings, where the aging population is growing most rapidly. In such populations, access to care for osteoporosis is limited, with many competing priorities for healthcare.^1^ There are few clinicians and common treatments for prevention of fracture are not included on the essential medicines list.^1^ In addition, access to imaging for vertebral fracture assessment (VFA) is limited and can be expensive, with radiographs being the most affordable technology. As with elsewhere on the globe, prevalence estimates vary, and methods of assessment and imaging often differ, as do the age ranges of inclusion.

VF are a major public health burden and are the commonest fragility fracture causing significant pain and mortality.^2^ VFs can be clinically challenging to detect and estimates range between 65% and 75% being undetected.^3^ This is due to the fractures often occurring asymptomatically and symptoms not manifesting until multiple fractures are present. In the UK, the 12-month survival rate in women aged 65 years and above was 86.5% compared to 93.6% expected, and a 5-yr survival rate of 56.5% compared to 69.9% expected.^4^ Importantly, VF are preventable and are risk factors for future VF and other major osteoporotic fractures. A limiting factor in determining the epidemiology of VF prevalence has been that there are several methods of assessment with lack of agreement on the best methodology to assess the presence or absence of fractures. In addition, incidental reporting on standard radiology is not common practice. Together, these make the comparison across populations difficult. In 2017, Ballane *et al.*,^5^ reported global prevalence data in men and women, with estimates ranging from 7% to 25%. Of note at the time, there was a lack of data from the Indian subcontinent, Middle East, or Africa, or globally across ethnic groups, indicative of disparities in healthcare and access to preventative treatment.

In South Africa, Conradie *et al.* reported VF prevalence in Black and White African women aged 40 yr and above, assessing lumbar spine and thoracic radiographs. Most fractures were classified as mild (60%), with a higher prevalence in Black (9.1%) vs White (5.0%) women.^6^ More recently, in older Black African and Indian South African adults from KwaZuluNatal, prevalence was reported to be higher than in the Conradie study at 20.8%, with no apparent difference between African and Indian participants.^7^ In Black women from the Republic of Congo, Central Africa, prevalence from CT scans was reported to be 11.2%.^8^ Currently, no data are available for incidence in Black African men and women and few data to describe risk factors for fracture with none in West Africa.

The primary aim of this study was to determine VF prevalence and risk factors for VF in men and women aged 40 yr and over in rural and urban regions of The Gambia, West Africa. Secondary aims were to calculate fracture incidence and associated risk factors for incident fractures in rural participants (who had been followed longitudinally).

## Materials and methods

### Participants

The Gambian Bone and Muscle Ageing Study (GamBAS-rural) is a prospective observational study in Black African men and women aged ≥40 yr (ISRCTN17900679). The study protocol has been published previously.^9^ In brief, rural participants were identified using the Kiang West Demographic Surveillance System (KWDSS).^9^^,^^10^ The target study sample for rural participants was 240 women and 240 men, with participants recruited and stratified by gender and by 5-yr age bands to ensure equal distribution. The oldest age band was 75+. Rural participants had baseline measurements in 2011-2012 and were followed up 6-8 yr later (2017-2019). A group of urban participants, aged 60-80 yr, were recruited in 2019 using the same inclusion criteria as GamBAS and had one assessment. Participants in GamBAS Urban were recruited through convenience sampling through community networks in Sukuta, West Coast Region, The Gambia. The ethnic group was self-reported. Assessments took place at the MRC Keneba and MRC Fajara bone imaging facilities. Ethics approval for all visits was granted by The Joint Gambia Government/MRC Unit The Gambia at London School of Hygiene and Tropical Medicine Ethics Committee (original reference SCC1222, new reference 28118).

### VFA and BMD Measurement

A GE-Lunar Prodigy Advance (GE-Lunar, Waltham, MA, USA, software version 15.0) was used to acquire baseline scans, and a GE-Lunar iDXA for longitudinal and urban scans (GE-Lunar, Waltham, MA, USA, software version 15.0). Scans of the lateral spine, proximal femur, lumbar spine, and whole body were acquired for measurement of areal BMD (aBMD); cross-calibration was performed between scanners.^11^ *T*- and *Z*-scores were calculated as per International Society of Clinical Densitometry (ISCD) guidelines, using National Health and Nutrition Examination Survey (NHANES) III data for *T*-score calculations and manufacturer reference for *Z*-scores.^12^ Body composition measurements were: total body fat mass (kg) and total body lean mass (kg). VFA scans were obtained to determine the presence of any spinal degeneration, by assessing vertebral shape, fracture, and osteoarthritic changes. The presence or absence of VF was determined at each vertebral level using the Genant semi-quantitative method, defining mild, moderate, and severe fractures based on reductions in vertebral anterior, middle, or posterior height/s.^13^

All scans were analyzed by a single expert reader (NJC) using version 18.0 of GE-iDXA software. Manufacturer standard procedures were followed for daily quality assurance and weekly quality control procedures. The same trained operators acquired scans across the two bone imaging facilities; the co-efficient of variation of repeat measurements in 30 Gambian adults was 0.7% at the total hip.

### Assessment of predictors of VF

#### Anthropometry

Baseline height (cm) was measured to the nearest 1 mm using a wall-mounted stadiometer (Seca GmbH, Hamburg, Germany) and weight (kg) measured to the nearest 0.1 kg using a digital scale (Seca GmbH, Hamburg, Germany) while the participants wore light clothing without footwear. BMI (kg/m^2^) was calculated by dividing weight by squared height. Similarly, fat mass (FMI) and lean mass indices (LMI) were calculated by dividing total body fat mass or total body lean mass by height squared.

#### Hand grip strength

Grip force (kg) was measured using a dynamometer (Jamar Hand Dynamometer, IL, USA).^14^ The individual was seated in an upright position with the dominant arm supported on the armrest of the chair, with the wrist in a neutral position, and the thumb facing upwards. Participants were instructed to exert maximal force. The maximum measurement of three test measurements was used for analysis (kg).

#### Lower limb muscle power

To assess lower limb muscle function, a Leonardo Ground Reaction Force Platform (Leonardo software version 4.2; Novotec Medical GmbH, Pforzheim, Germany) was used as described previously.^15–17^ Participants were asked to perform a jump as high as possible; this was repeated three times, and the maximum was taken for analysis.

#### Calcium intake

A prospective 2-d weighed dietary assessment was conducted in the participant’s home on days close to the measurement day by trained and experienced fieldworkers. This method was developed in and has been validated and used in previous studies in Kiang West with Gambian Food Tables being used to assess intake.^18^^,^^19^ Briefly, fieldworkers visited participants’ homes and recorded and weighed all food and drink items the participants consumed (total prepared minus amount left) over 48 hours. Data were coded and analyzed using the Diets-In, Nutrients-Out program with Gambian Food Tables.^20^^,^^21^

#### Biochemical assays

Blood samples were collected in lithium heparin (LH) and EDTA blood tubes from a forearm vein in the morning after an overnight fast. Plasma was separated by centrifugation at 1800 × *g* for 10 min at 4 °C, stored at −80 °C, and subsequently transported for analysis to the MRC Elsie Widdowson Laboratory, Cambridge, UK, on dry ice and stored at −80 °C. EDTA plasma was used for analysis of parathyroid hormone (PTH) and LH serum for bone turnover markers (procollagen type I N-terminal propeptide [P1NP], and serum collagen type 1 crosslinked β-C-telopeptide [β-CTx]) and 25-hydroxyvitamin D [25(OH)D]. Commercially available assay kits and platforms were used as follows for plasma: intact PTH, β-CTX, and P1NP were measured on the iSys platform (Immunodiagnostics Systems Ltd, Tyne and Wear, UK). For internal plasma drift control, NEQAS (Edinburgh, UK) was used for PTH and NEQAS IIA EQA (Sheffield, UK) for β-CTX and P1NP. 25(OH)D was analyzed in LH plasma using DiaSorin chemiluminescent immunoassay (Liaison; DiaSorin Inc., Stillwater, MN, USA) on an automated analyzer. Assay performance was monitored using kit and in-house controls and by participation in the Vitamin D External Quality Assessment Scheme (www.deqas.org). All assays performed well and were within specification (details are provided in Supplementary Information).

### Statistical analysis

Data analyses were conducted in Stata 17 (StataCorp, College Station, TX, USA). Normality of distribution of continuous variables was assessed graphically through histograms and were summarized using means and SDs, unless otherwise stated. Differences in continuous variables between fracture and non-fracture groups were compared using *t*-tests and those in categorical variables using chi-squared tests. Risk factors considered were anthropometric measures, bone turnover markers, PTH, 25(OH)D, grip strength, lower limb muscle power, mean calcium intake, and aBMD. Descriptive data are presented in all and by gender separately. For prevalence estimates and determination of risk factors, data for prevalent fracture data are analyzed by men and women separately. The prevalence of VF at baseline was calculated based on the number of individuals with at least one VF divided by the study population.^22^

In rural participants, we calculated the incidence of VFs as any new fracture identified at follow-up, irrespective of fracture status at baseline. The incidence was calculated overall and by sex. As sex-specific incidence was low, we combined and included sex as a potential risk factor.

Risk factors for prevalent and incident fractures were tested using univariable logistic regression, with and without adjustment for age and BMI. Data are presented as odds ratio (95% CI). No adjustments were made for multiple comparisons.

## Results

Prevalence of VFs was determined in 581 participants (298 women) at baseline (488 rural, 93 urban) (see Figure S1**)**. Of the 488 original rural participants, 281 were followed up in 2017-2019 (mean follow up time 7.2 SD yr). 214 of 281 participants scanned had available scans due to a database issue and formed the group in which incidence was determined.

Table 1 shows descriptive statistics of anthropometry, body composition, muscle function, and biochemistry for the whole group and by gender. All participants were Black African, being prominently of the Mandinka ethnic group (>90%).

**Table 1:** Baseline characteristics of cohort.

	**All**			**Men**			**Women**		
	**No fracture** **(*n* = 495)**	**Vertebral fracture** **(*n* = 86)**		**No fracture (*n* = 246)**	**Vertebral fracture (*n* = 37)**		**No fracture (*n* = 249)**	**Vertebral fracture (*n* = 49)**	
**Baseline age (y)**	61.7 (12.3)	65.6 (11.2)	0.006	62.1 (12.5)	64.3 (11.4)	0.313	61.4 (12.1)	66.7 (11.0)	0.005
**Area of residence^a^**									
** Urban (*n* (%))**	86 (17.4%)	12 (14.1%)	0.455	45 (18.4%)	4 (10.8%)	0.258	41 (16.5%)	8 (16.7%)	0.973
** Rural (*n* (%))**	408 (82.6%)	73 (85.9%)		200 (81.6%)	33 (89.2%)		208 (83.5%)	40 (83.3%)	
**Weight (kg)**	59.5 (12.5)	57.6 (13.4)	0.211	61.9 (12.3)	61.0 (13.2)	0.687	57.1 (12.2)	55.0 (13.2)	0.285
**Height (cm)**	163.4 (8.8)	162.9 (8.6)	0.639	169.2 (7.3)	169.6 (6.5)	0.783	157.7 (5.9)	157.8 (6.3)	0.906
**Body mass index (kg/m^2^)**	22.3 (4.3)	21.6 (4.4)	0.204	21.6 (3.7)	21.1 (4.1)	0.520	23.0 (4.7)	22.0 (4.6)	0.194
**Grip strength (kg)**	25.0 (9.4)	23.2 (8.5)	0.101	30.4 (9.4)	28.9 (8.5)	0.347	19.8 (5.8)	18.9 (5.6)	0.351
**Fat mass index (kg/m^2^)**	6.1 (3.4)	5.9 (3.5)	0.712	4.3 (2.3)	4.0 (2.7)	0.599	7.9 (3.3)	7.4 (3.4)	0.360
**Lean mass index (kg/m^2^)**	15.3 (2.0)	14.9 (2.1)	0.084	16.4 (1.8)	16.3 (1.9)	0.610	14.2 (1.7)	13.9 (1.5)	0.151
**2-leg jump power^b^ (Watt/kg)**	23.8 (10.8)	24.0 (11.7)	0.916	28.5 (12.1)	29.9 (12.7)	0.587	18.7 (6.1)	18.0 (6.9)	0.610
**Calcium intake (mg/d)**	342.0 (188.2)	333.2 (146.3)	0.712	383.7 (185.5)	358.3 (122.4)	0.463	302.5 (182.6)	312.6 (162.0)	0.751
**PTH^b^ (pg/mL)**	77.6 (33.1)	76.7 (35.8)	0.836	75.4 (32.8)	69.8 (33.2)	0.373	79.6 (33.4)	82.2 (37.2)	0.663
**Β-CTX^b^ (ng/mL)**	0.69 (0.32)	0.79 (0.36)	0.013	0.70 (0.32)	0.78 (0.35)	0.237	0.68 (0.31)	0.80 (0.36)	0.022
**P1NP^b^ (ng/mL)**	90.3 (38.9)	96.6 (43.9)	0.218	83.5 (31.3)	90.7 (35.1)	0.229	96.9 (44.0)	101.4 (49.9)	0.560
**25(OH)D^b^ (nmol/L)**	67.1 (18.5)	65.2 (19.6)	0.406	64.6 (18.2)	67.6 (21.4)	0.400	69.5 (18.5)	63.2 (17.9)	0.046

All continuous data are mean (SD) unless otherwise stated.

a Area of residence had two missing values.

b Data are only available in rural participants.

Abbreviations: PTH, parathyroid hormone; β-CTX, collagen type 1 crosslinked β-C-telopeptide; P1NP, procollagen type 1 N-terminal propeptide; 25(OH)D, 25-hydroxyvitamin D.

**Table 2:** Areal BMD in participants with and without vertebral fracture.

	**All**			**Men**			**Women**		
	**No fracture** **(*n* = 494)**	**Vertebral fracture** **(*n* = 84)**		**No fracture (*n* = 245)**	**Vertebral fracture (*n* = 36)**		**No fracture (*n* = 249)**	**Vertebral fracture (*n* = 48)**	
									
**Lumbar spine aBMD (g/cm^2^)**	0.97 (0.19)	0.85 (0.19)	<0.001	1.04 (0.17)	0.96 (0.18)	0.009	0.90 (0.19)	0.77 (0.15)	<0.001
**Lumbar spine *T*-score**	−2.14 (0.06)	−2.96 (0.15)	<0.001	−1.99 (0.07)	−2.36 (0.22)	0.082	−2.29 (0.10)	−3.42 (0.19)	<0.001
**Lumbar spine *Z*-score**	−1.21 (1.39)	−1.93 (1.33)	<0.001	−1.15 (1.40)	−1.75 (1.53)	0.030	−1.26 (1.37)	−2.06 (1.18)	<0.001
**Femoral neck aBMD (g/cm^2^)**	0.87 (0.15)	0.78 (0.15)	<0.001	0.91 (0.14)	0.86 (0.15)	0.088	0.83 (0.15)	0.73 (0.12)	<0.001
**Femoral neck *T*-score**	−1.24 (0.05)	−1.82 (0.11)	<0.001	−0.95 (0.06)	−1.26 (0.17)	0.082	−1.52 (0.07)	−2.25 (0.12)	<0.001
**Femoral neck *Z*-score**	−0.29 (0.99)	−0.74 (0.89)	<0.001	−0.32 (0.97)	−0.62 (0.98)	0.090	−0.26 (1.00)	−0.83 (0.80)	<0.001
**Total hip aBMD (g/cm^2^)**	0.91 (0.16)	0.82 (0.17)	<0.001	0.98 (0.14)	0.94 (0.15)	0.090	0.84 (0.16)	0.73 (0.13)	<0.001
**Total hip *T*-score**	−1.07 (0.05)	−1.73 (0.12)	<0.001	−0.83 (0.06)	−1.14 (0.18)	0.082	−1.30 (0.08)	−2.17 (0.14)	<0.001
**Total hip *Z*-score**	−0.32 (1.05)	−0.86 (1.03)	<0.001	−0.18 (1.01)	−0.51 (1.14)	0.075	−0.47 (1.07)	−1.13 (0.86)	<0.001

All continuous data are mean (SD) unless otherwise stated.

*T*-scores were derived using NHANES III and *Z*-scores using manufacturers references as per International Society for Clinical Densitometry guidance.

### VF prevalence

Prevalence of VF was 14.8% (95% CI: 12.01% − 18.0%); no differences were found in women compared to men (16.4% (95% CI: 12.4% − 21.1%) vs 13.1% (95% CI: 9.4% − 17.6%), *p* = .25). In participants aged 60 yr and over, it was 22.2%, in women (20.0%) and in men (16.3%). Figure S2 shows the distribution of fractures by severity. Most fractures were mild (62.8%), with the rest being moderate (31.4%) or severe (4.7%).

Eighty-six participants (49 women) had at least one prevalent VF; the total number of prevalent fractures was 138. Those with VF at baseline were older (65.6 vs 61.7 yr) than those without (*p*<.01). They were slightly leaner (mean (SD) LMI 14.9 (2.1) kg vs. 15.3 (2.0) kg in the whole group, *p* = .08) with no significant differences in body weight, BMI, or adiposity. Β-CTX was higher in those with a prevalent VF than in those without; this difference was more pronounced in women (*p* = .02). While overall, 25(OH)D was at sufficient levels by international standards, there was a trend for women with VFs to have lower plasma concentrations of 25(OH)D than those without fracture (63.2(17.9) vs 69.6 (18.5) nmol/L, *p* = .05).

**Figure 1:**
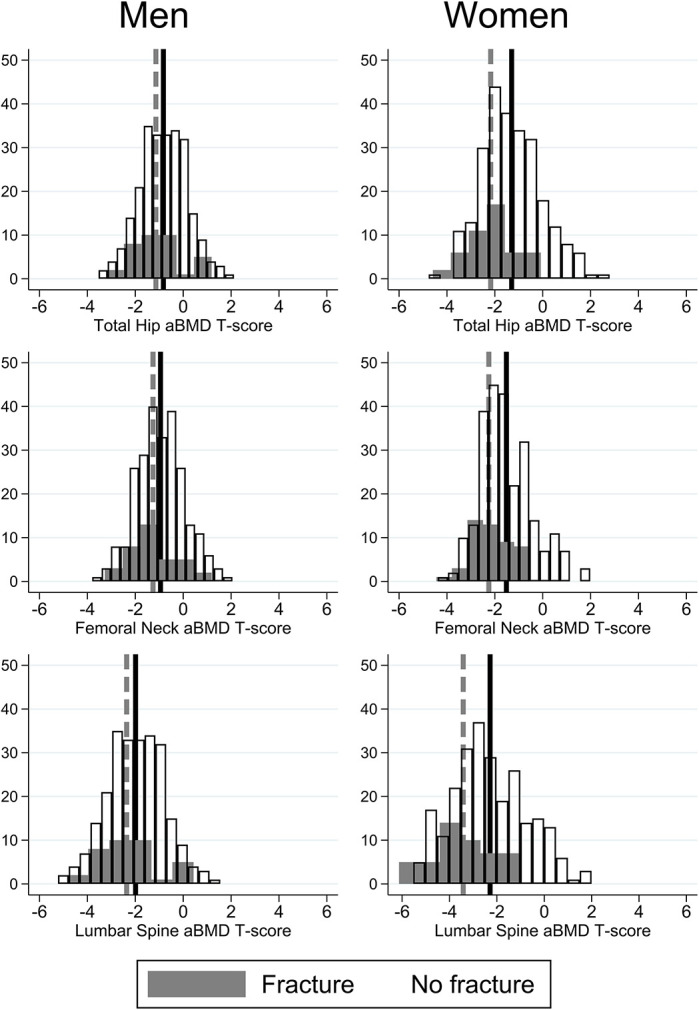
Frequency distribution of total hip, femoral neck, and lumbar spine *T*-scores by sex at baseline. Those with VF are in gray and dotted line, mean of non-fracture group are in white, with solid black line.

Table 2 presents the baseline DXA data. aBMD at the femoral neck, total hip, and lumbar spine was lower in the fracture compared to the no fracture group; in women and overall, this was statistically significant. In men, mean differences between groups were smaller, except for lumbar spine *T*-score (*p*-value = .01). Figure 1 shows *T*-score distributions in women and men separately. Lower femoral neck and L1-L4 *T*-score and *Z*-scores were also observed in those with VF compared to those without fracture; these differences were also observed in women, but only L1-L4 *T*-scores and *Z*-scores were significantly different in men (Table 2). In the whole population, osteoporosis prevalence was; 19.1% women, 6.0% men, 49.2% had osteopenia (53.4% women; 44.9% men). In those aged >60-years, 94.6% had a femoral neck *T*-score ≤ −2.5 and 76.9% a lumbar spine *T*-score  ≤ −2.5. In the 235 with information on VF and <60 years, 1.7% had osteoporosis (2.6% women; 0.9% men) and 34.2% had osteopenia (40.2% women; 28% men). In the 347 with information on VF and ≥60 years, 20.2% had osteoporosis (30.0% women; 9.6% men) and 59.4% had osteopenia (61.7% women; 56.9% men). Degenerative changes were reported in 10.8% of individuals at baseline and 13.3% at follow-up.

### Associations between risk factors and baseline prevalent fracture in rural and urban participants (Table 3)

For a 1 SD higher femoral neck *Z*-score, women were 48% less likely to have a prevalent VF (OR [95% CI]: 0.51 [0.34, 0.78], *p*<.001); similarly, for a 1SD higher total hip *Z*-score, they were 47% (0.53 [0.37, 0.77], *p*<.01) and 42% for a 1 SD higher lumbar spine *Z*-score (0.58 [0.42, 0.80], *p*<.01), less likely to have a prevalent VF. In men only lumbar spine *Z*-scores were predictive of prevalent VF; a 1SD higher *Z*-score associated with 34% decreased risk of fracture (0.66 [0.47, 0.93], *p* = .03).

**Table 3:** Baseline risk factors for prevalent vertebral fractures determined by logistic regression, unadjusted and adjusted for age and BMI. Data are presented as odds ratio (OR) (95% CI).

	**Women**				**Men**			
**Baseline determinants**		**Unadjusted**		**Adjusted for age and BMI**		**Unadjusted**		**Adjusted for age and BMI**
	***N***	**OR (95% CI)**	***N***	**OR (95% CI)**	***N***	**OR (95% CI)**	***N***	**OR (95% CI)**
**Urban (rural as reference)**	297	1.01 (0.44, 2.33)	296	1.03 (0.40, 2.68)	282	0.54 (0.18, 1.60)	282	0.45 (0.13, 1.50)
**Weight *Z*-score^a^^,^**	296	0.83 (0.60, 1.16)			281	0.93 (0.64, 1.33)	281	
**Height *Z*-score^a^^,^**	296	1.03 (0.65, 1.62)			281	1.06 (0.70, 1.61)	281	
**Max grip strength *Z*-score^b^**	296	0.79 (0.47, 1.30)	295	1.31 (0.69, 2.49)	281	0.84 (0.59, 1.20)	281	0.92 (0.58, 1.46)
**Lower limb muscle power *Z*-score ^b^**	188	0.82 (0.39, 1.75)	188	1.50 (0.59, 3.78)	201	1.11 (0.76, 1.61)	201	1.56 (0.91, 2.67)
**Total hip BMD *Z*-score^b^^,^^c^**	297	0.41 (0.28, 0.62)	296		281	0.70 (0.46, 1.06)	281	
**Femoral neck BMD *Z*-score^b^^,^^c^**	297	0.43 (0.29, 0.65)	296		281	0.71 (0.48, 1.05)	281	
**Lumbar spine BMD *Z*-score^b^^,^^c^**	297	0.43 (0.29, 0.63)	296		281	0.55 (0.35, 0.87)	281	
**M-Total hip *Z*-score^c^**	297	0.53 (0.38, 0.73)			282	0.73 (0.51, 1.03)		
**M-Femoral neck *Z*-score^c^**	297	0.51 (0.35, 0.73)			282	0.72 (0.49, 1.05)		
**M-Lumbar spine *Z*-score^c^**	277	0.61 (0.47, 0.80)			241	0.71 (0.53, 0.97)		
**Mean dietary calcium *Z*-score**	238	1.06 (0.75, 1.48)	238	1.06 (0.76, 1.48)	220	0.85 (0.56, 1.30)	220	0.87 (0.56, 1.34)
**PTH *Z*-score**	244	1.07 (0.78, 1.48)	244	0.97 (0.68, 1.39)	224	0.83 (0.54, 1.26)	224	0.76 (0.49, 1.19)
**Β-CTX *Z*-score**	247	1.43 (1.04, 1.95)	247	1.25 (0.89, 1.75)	230	1.23 (0.87, 1.73)	230	1.19 (0.84, 1.69)
**P1NP *Z*-score**	247	1.09 (0.82, 1.45)	247	1.01 (0.74, 1.37)	230	1.31 (0.84, 2.05)	230	1.33 (0.84, 2.10)
**25(OH)D *Z*-score**	247	0.68 (0.46, 0.99)	247	0.68 (0.47, 1.00)	230	1.17 (0.81, 1.67)	230	1.18 (0.82, 1.70)

a Weight and height *Z*-scores were not adjusted for BMI as weight and height are component parts of BMI calculation.

b *Z*-scores for exposures were calculated by individual value minus population mean, divided by population SD to allow comparison of effect size across exposures.

c BMD *Z*-scores are not adjusted for age as already expressed as a function of age. Abbreviations: M, manufacturer *Z*-score; PTH, parathyroid hormone; β-CTX, collagen type 1 crosslinked β-C-telopeptide; P1NP, Procollagen type 1 N-terminal propeptide; 25(OH)D, 25-hydroxyvitamin D.

In women, a SD increase in β-CTX was associated with a 40% higher likelihood of having a prevalent VF (1.43 [1.04, 1.95]), which was attenuated after adjustment for age and BMI 1.25 [0.89, 1.75]). A 1 SD increase in 25(OH)D was associated with 32% reduction in the likelihood of having a prevalent VF in women (*p* = .05) although this was attenuated after adjustment for age and BMI.

Area of residence, weight, height, BMI, P1NP, and PTH were not associated with prevalent VF in Gambian men and women.

### VF incidence in rural participants

Of 214 individuals with follow-up data 6-8 yr later, 180 had no prevalent VFs at baseline; there were 16 incident fractures (11 women, 5 men), while among the 34 with a prevalent VF at baseline, 7 women and 3 men had an incident fracture at another vertebral location at follow-up (10 fractures). Overall, this equates to an incidence of VFs in this population of 12.1%.

### Risk factors for incident fracture at follow-up (Table 4)

Similar risk factors were observed for incident fracture in the smaller sample of those successfully followed up (*n* = 26 incident fractures). For a 1 SD higher *Z*-score and after adjusting for age and BMI, the odds (OR[95% CI]) of having an incident VF reduced between 49% and 61% (femoral neck 0.51 [0.27, 0.98] *p* = .04, total hip 0.47 [0.28, 0.80] *p* = .01, lumbar spine 0.39 [0.21, 0.73], *p*<.001). A 1 SD reduction in grip strength *Z*-score was associated with a 47% reduction in the odds of having an incident fracture (0.53, (0.32, 0.88); adjustment for age and BMI attenuated this slightly (0.59, (0.34, 1.00), *p* = .05). Of the 34 people with prevalent fracture at baseline, 10 (29%) had a new fracture at follow-up; in comparison, 9% of those without fracture at baseline had an incident fracture.

**Table 4:** Baseline risk factors for incident vertebral fracture in rural participants, mean follow-up time 7.2 yr. Logistic regression unadjusted and adjusted for age and BMI. Data are presented as odds ratio (OR) (95% CI).

**Baseline determinant**	**Unadjusted**		**Adjusted for age**		**Adjusted for age and BMI**	
	**ORs (95% CI)**	***p*-value**	**ORs (95% CI)**	***p*-value**	**ORs (95% CI)**	***p*-value**
						
**Gender**	0.62 (0.25,1.50)	.29	0.66 (0.27,1.63)	.37	0.65 (0.26,1.60)	.34
**Weight *Z*-score^a^^,^^b^**	0.76 (0.45,1.29)	.31	0.88 (0.51,1.53)	.66		
**Height *Z*-score^a^^,^ ^b^**	0.80 (0.51,1.28)	.36	0.87 (0.54,1.41)	.57		
**Max grip strength *Z*-score^b^**	0.53 (0.32,0.88)	.01	0.59 (0.34,1.00)	.05	0.59 (0.34,1.00)	.05
**Lower limb muscle power *Z*-score^b^**	0.60 (0.35,1.03)	.06	0.71 (0.39,1.29)	.26	0.70 (0.38,1.29)	.25
**Total hip BMD *Z*-score^b^^,^^c^**	0.43 (0.26,0.72)	<.001				
**Femoral neck BMD *Z*-score^b^^,^^c^**	0.48 (0.28,0.81)	.01				
**Lumbar spine BMD *Z*-score^b^^,^^c^**	0.39 (0.23,0.66)	<.001				
**M_Total hip *Z*-score^c^**	0.45 (0.27,0.74)	<.001				
**M_Femoral neck *Z*-score^c^**	0.55 (0.32,0.94)	.03				
**M_L1-L4 *Z*-score^c^**	0.44 (0.27,0.72)	<.001				
**Mean dietary calcium *Z*-score^b^**	1.09 (0.69,1.71)	.72	1.03 (0.64,1.65)	.91	1.03 (0.64,1.65)	.92
**PTH *Z*-score^b^**	1.11 (0.77,1.60)	.59	1.03 (0.70,1.51)	.89	1.04 (0.70,1.54)	.85
**Β-CTX *Z*-score^b^**	1.38 (0.89,2.12)	.15	1.24 (0.78,1.96)	.36	1.23 (0.78,1.96)	.38
**P1NP *Z*-score^b^**	1.39 (0.97,1.99)	.07	1.32 (0.91,1.91)	.14	1.32 (0.91,1.91)	.14
**25(OH)D *Z*-score^b^**	0.95 (0.61,1.47)	.81	0.91 (0.59,1.41)	.68	0.90 (0.58,1.40)	.64

a Weight and height *Z*-scores were not adjusted for BMI as weight and height are component parts of BMI calculation.

b *Z*-scores for exposures were calculated by individual value minus population mean, divided by population SD to allow comparison of effect size across exposures.

c BMD *Z*-scores are not adjusted for age as already expressed as a function of age. Abbreviations: M, manufacturer *Z*-score; PTH, parathyroid hormone; β-CTX, collagen type 1 crosslinked β-C-telopeptide; P1NP, Procollagen type 1 N-terminal propeptide; 25(OH)D, 25-hydroxyvitamin D.

## Discussion

Given the importance of prevalent VFs in predicting future VFs and other fragility fractures in other populations, our findings are a major cause for concern in a population where resources are poor and fracture liaison services and prevention measures do not yet exist.^1^^,^^23^ Bisphosphonates and hormone replacement therapy are not currently on the World Health Organization Essential Medicines list for osteoporosis medication, limiting access to only private patients (in The Gambia, this is <3% of the general population).^1^^,^^23^ In this gender- and age-band stratified sample of men and women aged 40 yr and above, one in six individuals had at least one prevalent fracture. Women had more prevalent fractures than men, although the differences were not as large as those reported elsewhere (16% vs 13%); in women aged over 60-yr, 1 in 5 had at least one prevalent fracture. VF prevalence in The Gambia, one of the lowest income countries in the world (in 2022 gross domestic product per capita was 840 USD^24^), is therefore similar to higher-income countries.

Areal BMD and age were the strongest factors associated with VF. A 1 SD lower *Z*-score was associated with a 40%-50% greater chance of having at least one prevalent VF; in men, the relationship was not as strong with approximately 30% greater risk of prevalent fracture. Only a small proportion of individuals with VF (24.4%; 36.7% women, 8.1% men), would be diagnosed with osteoporosis using femoral neck *T*-score thresholds. In those under 60 years of age, only 2.6% women would be osteoporotic, and 1.0% men. In those aged over 60 years, the proportions are greater, 30.3% women, 9.6% men. More than half this population would be diagnosed with osteopenia (60.7% women; 56.9% men). Caution is therefore needed in attributing the VF prevalence reported in this study to osteoporosis. Men with VF were younger than women with VF. The younger age of men may indicate trauma-related fractures rather than fragility fractures *per se*, though this cannot be determined in the current study. In general, these findings show the use of a −2.5 SD *T*-score threshold, determined using NHANES III, cut-offs to distinguish fracture vs non-fracture cases is inappropriate. Furthermore, while the use of NHANES III is currently the only recommended and recognized way to diagnose osteoporosis and osteopenia in diverse populations, there is clearly a need to generate country-specific references for the creation of appropriate reference data curves.^1^ A further example of the limitation of using international references for diagnosis of osteoporosis was in a study of post-menopausal women in Zimbabwe, where racial and ethnic differences in body composition and body size were shown to impact diagnosis of osteoporosis.^25^

Cross-country comparison of VF prevalence is difficult given differences in imaging technology and population sampling. In South Africa, prevalence in Black and Indian African men and women, median age 72.0 yr, was 20.8% in women, 13.0% in men.^7^ Women were twice as likely to have a prevalent VF as men. These more recent data from South Africa are more than twice the prevalence previously described in Black and White South African women aged 40 yr and over, where they were reported to be 9.0% and 5.1%, respectively. The data potentially indicate increasing VF prevalence, though the differences in the age range and methodologies of the respective study populations should be noted.^6^ In the Republic of the Congo in Central Africa, prevalence was reported to be 11.2% in women aged 40 yr and above, though it should be noted that these assessments were using reformatted CT scans and the women were mostly in high socioeconomic classes of the country.^8^ The recent South African study is the only other African study to present aBMD in individuals with and without VFs. Lumbar spine aBMD was lower in those with fractures, consistent with our findings in GamBAS though in contrast to GamBAS there were no differences in femoral neck or total hip aBMD. No other risk factors differed, though the sample size was limited, which may have impacted results.

Understanding the underlying causes and potential risk factors for VFs is important to address the need for prevention of future fragility fractures. In our study, age was higher and aBMD lower, and consequently *Z*-scores were lower, in those with fractures than those without. Women with VFs had lower 25(OH)D (63.2 nmol/L) compared to those without (69.5 nmol/L) with a trend toward lower 25(OH)D in women being risk factors for fracture. Despite 25(OH)D being adequate, compared to international standards, there is a possibility that in a population with ubiquitous UVB sunshine, the contribution of low dietary calcium intakes leads to skeletal sequalae at higher 25(OH)D concentrations and/or differing thresholds for sufficiency.^26^ Although habitual calcium intake was very low relative to international standards,^27^ calcium intake was not associated with increased risk for fracture. In addition, there was no evidence that BMI, FMI, and LMI were factors associated with prevalent VF. One potential explanation for the lack of association with BMI, lean, or fat mass is that the study population was relatively homogenous, with few people with high BMI. While incident VF numbers were low, bone turnover was also associated with increased risk of incident VF. P1NP associations were robust to adjustment for age, whereas CTX were not. There was a trend toward baseline grip strength being predictive of incident VF, albeit attenuated by adjustment for age, potentially reflecting the frailty status of the participants with fractures.

The implications of these findings are several fold. In a cohort of older Gambia adults, the prevalence of VFs was similar to that reported elsewhere in the world.^5^ Second, findings demonstrate the importance of not assuming risk factors translate across contexts. As the number of older individuals living in low- and middle-income countries increases exponentially, strategies to identify, prevent, and treat such fractures become even more important.^28^ In these resource-poor settings, access to anti-resorptive therapies, expertise in bone health, fracture liaison services, and hormone replacement therapy is limited. Therefore, understanding better the risk factors for fracture and seeking implementable ways of identifying VF and predicting the risk of future VF, with context-specific primary and secondary prevention strategies is paramount. More broadly, facilitating community knowledge and care is also an extremely important aspect of addressing the challenge posed by rising numbers of the aging population.

### Strengths and limitations

The rural component of this work is one of the most detailed, prospective musculoskeletal phenotyping in aging adults in Africa to date. This is strengthened by study design, which ensured, through use of the Kiang West Demographic Surveillance Survey,^10^ random stratified sampling in 5-yr age bands and by gender, ensured rural participants were distributed appropriately across the region. All scans were read and analyzed by a single expert reader. Cross-calibrations for aBMD and body composition measured on the different instruments were performed, making all rural and urban measurements directly comparable.^11^ The addition of urban subjects, prominently of the same ethnic group as the rural sample (Mandinka), allowed comparison of prevalence between urbanized and rural sites in The Gambia.

There are some limitations to this study. Findings may not be generalizable to the whole population; the focus here was on two regions (Sukuta is one of the most densely populated urban conurbations of the Gambia). GamBAS was powered to detect change in total hip aBMD, not to ascertain VF prevalence or incidence, or the associated risk factors. The number of incident fractures was low, making the generalizability of findings uncertain; more adequately powered studies are required. Second, DXA equipment was upgraded mid-study. Urban recruitment was at a different time to rural recruitment. Methods to recruit differed due to a lack of Demographic Surveillance System in Urban Gambia meaning convenience sampling was not used. Older adults were weighted toward older ages (60-80 yr), making the mean age slightly older than in the rural region. In addition. Some risk factor data were not available for the urban sample, limiting power to detect associations and to predict incident fracture. Self-reported fracture data were incomplete, meaning the association between pervious fracture and prevalent VF could not be tested. We did not adjust for multiple comparisons and cannot rule out findings that may have been due to chance, though the analyses performed were based on a priori hypotheses formed by known associations between age, BMD, body composition, and bone turnover on bone. Setting more conservative thresholds for significance may have increased the likelihood of missing true associations.

## Conclusions

In conclusion, contrary to common perception, VF prevalence in Gambian women and men is similar to that seen in higher-income countries across the world. Given the rising aging population in low- and middle-income countries, and the consequent rises in non-communicable diseases, such as osteoporosis, this is concerning. There are currently no treatment options for VFs apart from pain relief. Finding scalable and achievable ways to inform communities and healthcare professionals of fractures and their consequences is of utmost important to prevent further challenges to already stretched healthcare resources and, most importantly, to individuals themselves.

## Supplementary Material

Supplementary_Figure_S1_zjae182

Supplementary_figure_2_zjae182

Supplementary_information_05-11-24_zjae182

## Data Availability

The data that support the findings of this study are available from the corresponding author upon reasonable request.

## Study registration

ISRCTN17900679.
