# Correction to: Vertebral fracture prevalence and risk factors for fracture in The Gambia, West Africa: the Gambian Bone and Muscle Ageing Study

**DOI:** 10.1093/jbmr/zjaf109

**Published:** 2025-09-30

This is a correction to: Kate A Ward, Landing Jarjou, Camille Pearse, Mícheál Ó Breasail, Ramatoulie E Janha, Ayse Zengin, Ann Prentice, Nicola J Crabtree, Vertebral fracture prevalence and risk factors for fracture in The Gambia, West Africa: the Gambian Bone and Muscle Ageing Study, *Journal of Bone and Mineral Research*, Volume 40, Issue 1, January 2025, Pages 50–58, 10.1093/jbmr/zjae182

In the originally published manuscript, the incorrect equation was applied to calculate T-scores from NHANES III references. The wrong manufacturer equation was used, applying the Hologic standardization equation to transform data (which should be applied to data acquired on a Hologic scanner) rather than GE-Lunar to standardize the BMD. There was therefore an underestimation of the proportions of people with osteoporosis and osteopenia. All relevant results are emended in the main text, Figure 1 and Table 2. This change shifts the distribution of T-scores to the negative; this doesn't change any of the univariate or multivariate analysis as the magnitude of the differences remains the same.

GE-Lunar BMD has been standardized to Hologic, meaning measurements are made equivalent to if they were measured on a Hologic scanner. The equation applied is: Hologic equivalent T_score = (GE-Lunar FNBMD - 1.038)/0.139

Therefore, if GE-Lunar measurements are standardized to Hologic, they will become lower meaning the T-scores would be lower and shifting the T-score distribution to the negative. This is consistent with the observations of Analay et al. showing that GE-Lunar measures higher than Hologic.

The approach taken is consistent with International Society for Clinical Densitometry guidelines and as shown in Kanis et al. (Osteoporos Int, 24(11).

**Page 54**: The prevalence of osteoporosis has been updated throughout. Table 2 has been updated.

**Pages 55-56**

1. The prevalence of individuals with VF diagnosed with osteoporosis has been added, to give clarity to the statement.

2. The initial wording discussed very low prevalence of osteoporosis in men and women aged below and over 60 years of age. Because the prevalence is now higher (due to recalculation of the T-scores) the sentence has been edited.

3. The initial wording regarding osteopenia has been updated to give the new figures.

Figure 1 shows the T-score distribution of the population with and without prevalent fracture. The update is required because the T-scores were calculated incorrectly. The distributions are the same but mean T-scores shifted to the negative.

Figure 1 should read:


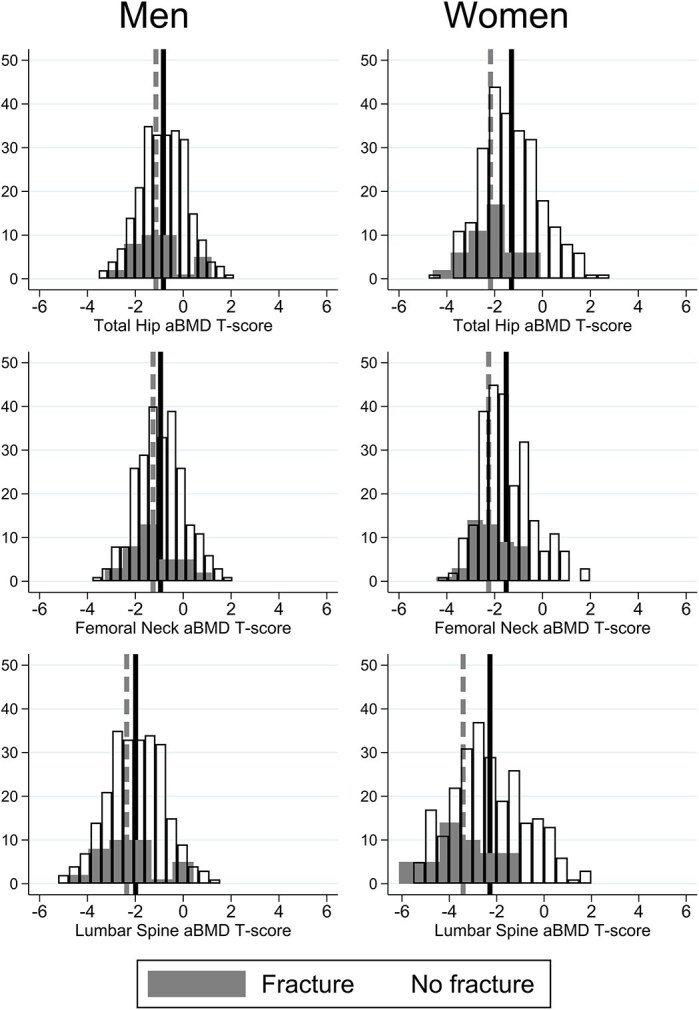


instead of:


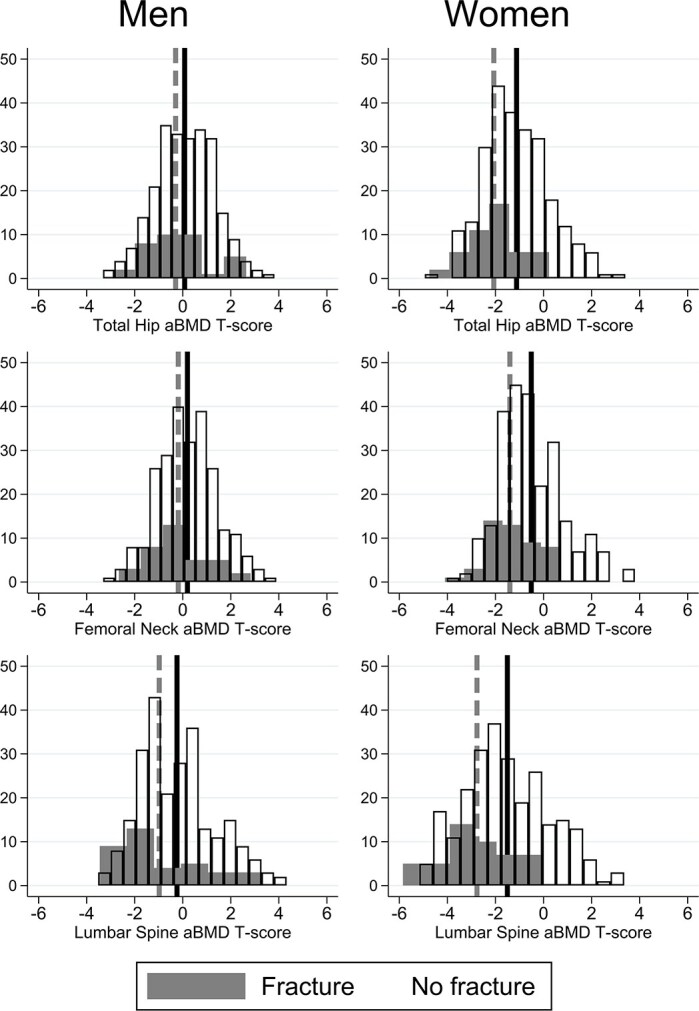


The T-scores descriptives in Table 2 have been updated accordingly.

Table 2 should read:

**Table 2:** Areal bone mineral density in participants with and without vertebral fracture.

	**All**			**Men**			**Women**		
	**No fracture** **(*n* = 494)**	**Vertebral fracture** **(*n* = 84)**		**No fracture (*n* = 245)**	**Vertebral fracture (*n* = 36)**		**No fracture (*n* = 249)**	**Vertebral fracture (*n* = 48)**	
									
**Lumbar spine aBMD (g/cm^2^)**	0.97 (0.19)	0.85 (0.19)	<0.001	1.04 (0.17)	0.96 (0.18)	0.009	0.90 (0.19)	0.77 (0.15)	<0.001
**Lumbar spine *T*-score**	−2.14 (0.06)	−2.96 (0.15)	<0.001	−1.99 (0.07)	−2.36 (0.22)	0.082	−2.29 (0.10)	−3.42 (0.19)	<0.001
**Lumbar spine *Z*-score**	−1.21 (1.39)	−1.93 (1.33)	<0.001	−1.15 (1.40)	−1.75 (1.53)	0.030	−1.26 (1.37)	−2.06 (1.18)	<0.001
**Femoral neck aBMD (g/cm^2^)**	0.87 (0.15)	0.78 (0.15)	<0.001	0.91 (0.14)	0.86 (0.15)	0.088	0.83 (0.15)	0.73 (0.12)	<0.001
**Femoral neck *T*-score**	−1.24 (0.05)	−1.82 (0.11)	<0.001	−0.95 (0.06)	−1.26 (0.17)	0.082	−1.52 (0.07)	−2.25 (0.12)	<0.001
**Femoral neck *Z*-score**	−0.29 (0.99)	−0.74 (0.89)	<0.001	−0.32 (0.97)	−0.62 (0.98)	0.090	−0.26 (1.00)	−0.83 (0.80)	<0.001
**Total hip aBMD (g/cm^2^)**	0.91 (0.16)	0.82 (0.17)	<0.001	0.98 (0.14)	0.94 (0.15)	0.090	0.84 (0.16)	0.73 (0.13)	<0.001
**Total hip *T*-score**	−1.07 (0.05)	−1.73 (0.12)	<0.001	−0.83 (0.06)	−1.14 (0.18)	0.082	−1.30 (0.08)	−2.17 (0.14)	<0.001
**Total hip *Z*-score**	−0.32 (1.05)	−0.86 (1.03)	<0.001	−0.18 (1.01)	−0.51 (1.14)	0.075	−0.47 (1.07)	−1.13 (0.86)	<0.001

All continuous data are mean (SD) unless otherwise stated.

*T*-scores were derived using NHANES III and *Z*-scores using manufacturers references as per International Society for Clinical Densitometry guidance.

instead of:

**Table 2:** Areal BMD in participants with and without vertebral fracture.

	**All**			**Men**			**Women**		
	**No fracture** **(*n* = 494)**	**Vertebral fracture** **(*n* = 84)**		**No fracture (*n* = 245)**	**Vertebral fracture (*n* = 36)**		**No fracture (*n* = 249)**	**Vertebral fracture (*n* = 48)**	
									
**Lumbar spine aBMD (g/cm^2^)**	0.97 (0.19)	0.85 (0.19)	<0.001	1.04 (0.17)	0.96 (0.18)	0.009	0.90 (0.19)	0.77 (0.15)	<0.001
**Lumbar spine *T*-score**	−0.88 (1.79)	−1.99 (1.80)	<0.001	−0.24 (1.57)	−0.98 (1.71)	0.009	−1.50 (1.78)	−2.77 (1.46)	<0.001
**Lumbar spine *Z*-score**	−1.21 (1.39)	−1.93 (1.33)	<0.001	−1.15 (1.40)	−1.75 (1.53)	0.030	−1.26 (1.37)	−2.06 (1.18)	<0.001
**Femoral neck aBMD (g/cm^2^)**	0.87 (0.15)	0.78 (0.15)	<0.001	0.91 (0.14)	0.86 (0.15)	0.088	0.83 (0.15)	0.73 (0.12)	<0.001
**Femoral neck *T*-score**	−0.1 6 (1.33)	−0.88 (1.29)	<0.001	0.19 (1.22)	−0.19 (1.29)	0.088	−0.51 (1.34)	−1.40 (1.04)	<0.001
**Femoral neck *Z*-score**	−0.29 (0.99)	−0.74 (0.89)	<0.001	−0.32 (0.97)	−0.62 (0.98)	0.090	−0.26 (1.00)	−0.83 (0.80)	<0.001
**Total hip aBMD (g/cm^2^)**	0.91 (0.16)	0.82 (0.17)	<0.001	0.98 (0.14)	0.94 (0.15)	0.090	0.84 (0.16)	0.73 (0.13)	<0.001
**Total hip *T*-score**	−0.52 (1.44)	−1.31 (1.50)	<0.001	0.08 (1.23)	−0.30 (1.36)	0.090	−1.12 (1.38)	−2.07 (1.10)	<0.001
**Total hip *Z*-score**	−0.32 (1.05)	−0.86 (1.03)	<0.001	−0.18 (1.01)	−0.51 (1.14)	0.075	−0.47 (1.07)	−1.13 (0.86)	<0.001

All continuous data are mean (SD) unless otherwise stated.

*T*-scores were derived using NHANES III and *Z*-scores using manufacturers references as per International Society for Clinical Densitometry guidance.

The emendations have been made within the article.

