# Supplementary figures and images for: Vertebral fracture prevalence and risk factors for fracture in The Gambia, West Africa: the Gambian Bone and Muscle Ageing Study
Source: J Bone Miner Res. 2024 Nov 7;40(1):50–8. doi: 10.1093/jbmr/zjae182 (PMC11700582; doi:10.1093/jbmr/zjae182)

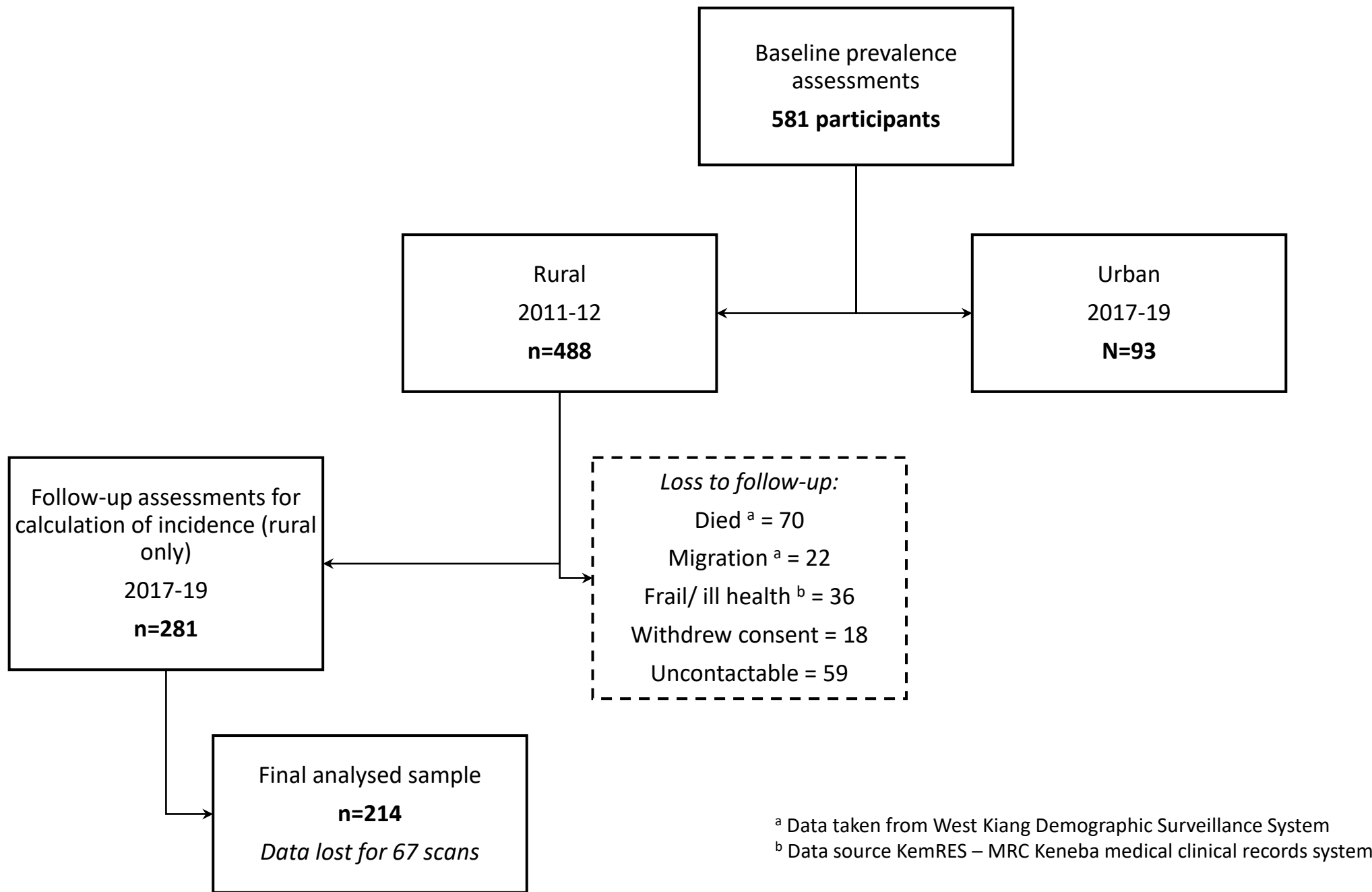

Supplement: Supplementary_Figure_S1_zjae182 [file Supplementary_Figure_S1_zjae182.pdf]
