# Supplementary material for: Vertebral fracture prevalence and risk factors for fracture in The Gambia, West Africa: the Gambian Bone and Muscle Ageing Study
Source: J Bone Miner Res. 2024 Nov 7;40(1):50–8. doi: 10.1093/jbmr/zjae182 (PMC11700582; doi:10.1093/jbmr/zjae182)

**Supplementary Figure 2**: Frequency by location and severity of fracture as per Genant semi-quantitative method


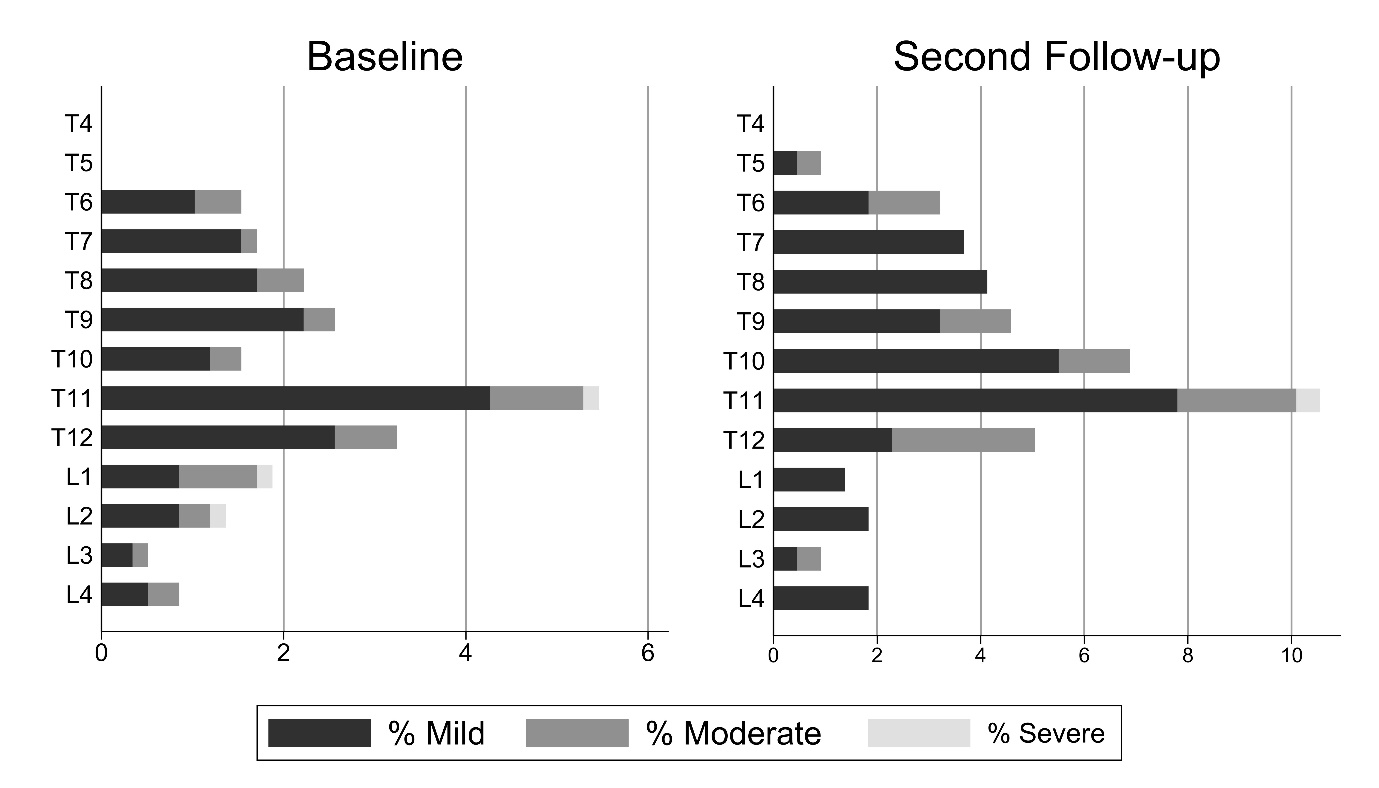

Supplement: Supplementary_figure_2_zjae182 [file Supplementary_figure_2_zjae182.docx]
