# Supplementary material for: Vertebral fracture prevalence and risk factors for fracture in The Gambia, West Africa: the Gambian Bone and Muscle Ageing Study
Source: J Bone Miner Res. 2024 Nov 7;40(1):50–8. doi: 10.1093/jbmr/zjae182 (PMC11700582; doi:10.1093/jbmr/zjae182)
Supplement: Supplementary_information_05-11-24_zjae182 [file Supplementary_information_05-11-24_zjae182.docx]

**Supplementary data:**

**QC reports for biochemical assays – all analyses were performed at MRC Human Nutrition Research Unit, Cambridge, United Kingdom**

Intact PTH, β-CTX and P1NP were measured on the iSys platform (Immunodiagnostics Systems Ltd, Tyne and Wear, UK). For internal plasma drift control: NEQAS (Edinburgh, UK) was used for PTH and NEQAS IIA EQA (Sheffield, UK) for β-CTX and P1NP.

***Parathyroid hormone.***

Assay range low – 40-60pg/ml, high – 322-466 pg/ml

| **Run No** | **Plot No** | **Kit-Low pg/ml** | **%CV** | **Kit High pg/ml** | **%CV** | **EDTA 11 pg/ml** | **%CV** |  |
| --- | --- | --- | --- | --- | --- | --- | --- | --- |
|  |  |  |  |  |  |  |  |  |
| 1 | 1 | 47.90 | 9.15 | 360.00 | 0.79 | 41.05 | 2.93 |  |
|  | 2 | 50.30 | Singleton | 377.00 | Singleton |  |  |  |
| 2 | 3 | 48.45 | 8.03 | 377.00 | 1.50 | 40.60 | 11.15 |  |
|  | 4 | 49.60 | Singleton | 345.00 | Singleton | 43.80 | Singleton |  |
| 3 | 5 | 47.00 | 2.11 | 367.50 | 5.96 | 39.25 | 5.22 |  |
|  | 6 | 44.90 | Singleton | 337.00 | Singleton |  |  |  |

***Procollagen type I N-terminal propeptide [P1NP]***

Assay range low – 30-51 µg/L, high – 322-466 µg/L

|  | |  |  | **97-150** |  |  |  |  |
| --- | --- | --- | --- | --- | --- | --- | --- | --- |
| **Run No.** | **Plot No** | **Kit-Low µg/L** | **%CV** | **Kit-High µg/L** | **%CV** | **PQC µg/L** | **%CV** |  |
|  |  |  |  |  |  |  |  |  |
| 1 | 1 | 42.12 | 0.24 | 132.286 | 1.46 | 50.82 | 1.15 |  |
|  |  |  |  |  |  | 51.10 | 1.34 |  |
| 2 | 2 | 38.40 | 0.41 | 130.52 | 0.57 | 47.89 | 1.33 |  |

***Serum collagen type 1 crosslinked β‐C‐telopeptide [β‐CTx])***

Assay range low – 0.230-0.344ng/L, high 0.732-1.098ng/ml

|  | |  |  |  |  | |  |  |  |  |
| --- | --- | --- | --- | --- | --- | --- | --- | --- | --- | --- |
| **Run No** | **Plot No** | **Kit - Low ng/ml** | **%CV** | **Kit - High ng/ml** | **%CV** | **SBTS ng/ml** | **%CV** | **PQC ng/ml** | **%CV** |  |
|  |  |  |  |  |  |  |  |  |  |  |
| 1 | 1 | 0.30 | 3.02 | 0.88 | 2.34 | 0.34 | 1.46 | 0.31 | 3.20 |  |
| 2 | 2 | 0.29 | 1.71 | 0.90 | 6.14 | 0.38 | 0.33 | 0.33 | 2.82 |  |
| 3 | 3 | 0.30 | Singleton | 0.91 | 4.83 | 0.35 | 1.74 | 0.33 | 2.33 |  |

***25-hydroxy vitamin D***

*Analysis by Diasorin*

| Range | **24.3-51.5** | | **86.5-160.5** | | **32.9 - 43.9** | | **38.2-70.9** |  | **72.1-134** |  |  |
| --- | --- | --- | --- | --- | --- | --- | --- | --- | --- | --- | --- |
| **Run No** | **Kit-Low nmol/L** | **%CV** | **Kit-High nmol/L** | **%CV** | **SBTS nmol/L** | **%CV** | **Liquicheck-1** | **%CV** | **Liquicheck-2** | **%CV** |  |
|  |  |  |  |  |  |  |  |  |  |  |  |
| 1 | 38.20 | 1.85 | 129.00 | 2.19 | 38.65 | 5.31 | 52.45 | 3.37 | 90.75 | 3.66 |  |
|  | 41.00 | 8.28 | 136.50 | 4.66 | 37.50 | Singlet |  |  |  |  |  |
|  | 39.65 | 1.25 | 123.50 | 6.30 | 37.45 | 2.83 |  |  |  |  |  |
| 2 | 37.10 | 2.29 | 126.00 | 1.12 | 39.00 | 3.99 | 53.75 | 2.24 | 91.65 | 0.85 |  |
|  | 39.20 | 6.49 | 130.50 | 5.96 | 36.40 | 1.17 |  |  |  |  |  |
|  | 39.75 | 1.96 | 124.50 | 1.70 | 36.55 | 4.84 |  |  |  |  |  |
